# A Text Book of Pathological Anatomy and Pathogenesis

**Published:** 1887-09

**Authors:** 


**A Text-Book of Pathological Anatomy and Pathogenesis.** By ERNST ZIEGLER, Professor of Pathological Anatomy in the University of Tubingen. Translated and edited for English students by Donald MacAlister, M. A., M. D., F. R. C. P., University Lecturer on Medicine, etc., etc. Three parts complete in one volume. New York: Wm. Wood & Co. 1887.

It would almost seem that every good book, published in whatever tongue, will sooner or later be offered to American readers by these indefatigable publishers; and not only so, but translations bearing the imprint of Wood & Co. are commonly much better books than the originals. The present one, for instance, has been wisely edited and translated by Dr. MacAlister, and additions have been made even to the last German edition, which increases greatly the value of the book to American readers. The book, originally issued in three parts, now makes a handsome volume of over 1,000 pages. The illustrations are excellent. In its present form, the work will be found most valuable by all English and American workers in pathology.

